# Evaluation of mulberry leaves’ hypoglycemic properties and hypoglycemic mechanisms

**DOI:** 10.3389/fphar.2023.1045309

**Published:** 2023-04-06

**Authors:** Sikai Chen, Miaomiao Xi, Feng Gao, Min Li, TaiWei Dong, Zhixin Geng, Chunyu Liu, Fengyu Huang, Jing Wang, Xingyu Li, Peifeng Wei, Feng Miao

**Affiliations:** ^1^ Shaanxi University of Chinese Medicine, Xianyang, China; ^2^ The Second Affiliated Hospital of Shaanxi University of Chinese Medicine, Xianyang, China; ^3^ Xi’an TANK Medicinal Biology Institute, Xi’an, China

**Keywords:** mulberry leaves, chemical substances in mulberry leaves, mulberry leaves safety, diabetes treatmen, molecular mechanism

## Abstract

The effectiveness of herbal medicine in treating diabetes has grown in recent years, but the precise mechanism by which it does so is still unclear to both medical professionals and diabetics. In traditional Chinese medicine, mulberry leaf is used to treat inflammation, colds, and antiviral illnesses. Mulberry leaves are one of the herbs with many medicinal applications, and as mulberry leaf study grows, there is mounting evidence that these leaves also have potent anti-diabetic properties. The direct role of mulberry leaf as a natural remedy in the treatment of diabetes has been proven in several studies and clinical trials. However, because mulberry leaf is a more potent remedy for diabetes, a deeper understanding of how it works is required. The bioactive compounds flavonoids, alkaloids, polysaccharides, polyphenols, volatile oils, sterols, amino acids, and a variety of inorganic trace elements and vitamins, among others, have been found to be abundant in mulberry leaves. Among these compounds, flavonoids, alkaloids, polysaccharides, and polyphenols have a stronger link to diabetes. Of course, trace minerals and vitamins also contribute to blood sugar regulation. Inhibiting alpha glucosidase activity in the intestine, regulating lipid metabolism in the body, protecting pancreatic -cells, lowering insulin resistance, accelerating glucose uptake by target tissues, and improving oxidative stress levels in the body are some of the main therapeutic properties mentioned above. These mechanisms can effectively regulate blood glucose levels. The therapeutic effects of the bioactive compounds found in mulberry leaves on diabetes mellitus and their associated molecular mechanisms are the main topics of this paper’s overview of the state of the art in mulberry leaf research for the treatment of diabetes mellitus.

## 1 Introduction

Diabetes mellitus (DM), a metabolic disorder that causes polyphagia, polydipsia, polyuria, and weight loss clinically, is characterized by an absolute or relative lack of insulin secretion and decreased sensitivity of target organs to insulin. It is estimated that by 2045, the number of people with diabetes will reach 783.2 million worldwide (Rosengren and Dikaiou, 2023). A study from China shows that the number of people with diabetes in the country will reach 217.64 million by 2030 (Sun et al., 2022). The global health problems caused by diabetes are on the rise. High blood glucose levels brought on by DM, a metabolic disorder, can result in major consequences and permanent harm to the cardiovascular system, eyes, and limbs (Zimmet et al., 2018). In the annals of Chinese and global medicine and culture, TCM is a gem. It has an impact not just in Asia but also in some other regions where the concept of TCM is progressively becoming embraced by the international medical community (Bauer, 2015). The globe will thereby benefit from traditional Chinese medicine’s fuller approach to the diabetes problem and patients’ quality of life. Chinese herbs Dendrobium, Ginkgo Biloba, Rhodiola Rosea, Cornu Cervi Pantotrichum, and Mulberry Leaf are all good at regulating blood sugar (Sun et al., 2021). In traditional Chinese medicine as well as modern Chinese and Western medicine, mulberry leaves’ active ingredients are frequently used to treat metabolic diseases like diabetes. The effect of mulberry leaves has been well-documented in ancient China for its capacity to dissipate wind-heat, clear the lungs, and moisten dryness (Wang et al., 2018; Hao Y. et al., 2021). Mulberry leaf therapy for treating diabetes is therefore very promising and should be thoroughly researched.

Mulberry leaves are the dried leaves of the mulberry (Morusalba L.), a plant of the mulberry family, which is widely distributed in tropical, subtropical and temperate regions (Agarwal and Kanwar, 2007). Modern studies have demonstrated that the active plant substances flavonoids, alkaloids, polysaccharides, and polyphenols are the primary molecular basis of mulberry leaves’ ability to lower blood sugar (Ge et al., 2018). Moderate supplementation of flavonoids in the diet can enhance pancreatic β-cell viability, reduce insulin resistance, and improve lipid and inflammatory responses (van Dam et al., 2013). In the history of human medication use for the treatment and prevention of disease, alkaloids are without a doubt one of the more ancient naturally occurring active chemicals. Many plants contain alkaloids, including Capsicum, Turmeric, and Berberis. Researchers have extracted alkaloids from these plants for experimental studies and clinical research, and they discovered that the natural alkaloids they contain are better for controlling blood glucose, an effect connected to the alkaloids’ capacity to inhibit α-glucosidase, increase insulin sensitivity, and control oxidative stress in the body (Ajebli et al., 2021). Scientists have discovered that polysaccharides occur in cell membranes and play crucial physiological roles in processes including anti-oxidation, blood sugar management, and immunomodulation in addition to being a part of the energy source in living things. One of the most crucial active substances in Chinese medicine for the treatment of diseases is polysaccharides, which can more visibly control blood sugar and anti-diabetes (Wu et al., 2016). Herbal polysaccharides’ ability to lower pancreatic β-cells apoptosis and increase glucose uptake may be how they work to regulate blood sugar levels (Zheng et al., 2019). Polyphenols are also important in maintaining blood glucose homeostasis, and studies have shown that adequate intake of polyphenols in the daily diet can have a beneficial effect on balancing blood glucose, possibly through anti-inflammatory, antioxidant and glucose absorption in the intestine (Kim et al., 2016).

This essay will examine how mulberry leaves’ flavonoids, alkaloids, polysaccharides, and polyphenols lower blood sugar levels. This article will go over more elements and metabolic pathways in mulberry leaves that lower glucose. It is intended that this page can help researchers design, investigate, and create medications based on mulberry leaves.

## 2 Main bioactive components of mulberry leaves

### 2.1 Flavonoid compounds

Mulberry leaves can be found in many different countries around the world, and because the cultivars growing there vary, so do the flavonoids the leaves contain. Additionally, flavonoids are determined using a variety of methodologies, and measurement methods and procedures are constantly being improved. Due to this, the flavonoids discovered in mulberry leaves during the past 20 years have been compiled. Because of the large number of flavonoids contained in mulberry leaves, the article expresses the categories of flavonoids contained in its mulberry leaves in the body of the article. The names of the specific flavonoids involved are described in Table 1. Mulberry leaves contain a large number of flavonoids, which can be broadly classified into the following groups of components. Quercetin, isoquercitrin and quercetin glycosides with quercetin as the parent nucleus, kaempferol and kaempferol glycosides with kaempferol as the parent nucleus, rutin-like compounds, lignan and lignan glycosides with lignan as the parent nucleus, morusin, sanggenon, morin, astrogaloside and other types of flavonoids found in mulberry leaves (Kim et al., 1999; Doi et al., 2001; Dat et al., 2010; Yang et al., 2010; Kim and Jang, 2011; Hong et al., 2013; Araujo et al., 2015; Chan et al., 2016; Hao et al., 2018; Thaipitakwong et al., 2018; Han et al., 2020; Niu et al., 2020; Hao M. et al., 2021).

**TABLE 1:** Main chemical composition of mulberry leaves.

NO	Flavonoids	References
1	quercetin 3-(6-malonylglucoside)	Chan et al. (2016)
2	rutin	Chan et al. (2016)
		Hao et al. (2018)
		Kim and Jang (2011)
3	isoquercitrin	Chan et al. (2016)
		Han et al. (2020)
4	isoprenoid flavan	Doi et al. (2001)
5	3′-geranyl-3-isoprenoid-2′,4′,5,7-tetrahydroxyflavone	Dat et al. (2010)
6	3′,8-diisoprenoid-4′,5,7-trihydroxyflavone	Dat et al. (2010)
7	kuwanon S	Dat et al. (2010)
8	8-geranylapigenin	Dat et al. (2010)
9	kaempferol	Dat et al. (2010)
10	morusin	Dat et al. (2010)
11	sanggenon J	Dat et al. (2010)
12	sanggenon K	Dat et al. (2010)
13	cyclomorusin	Dat et al. (2010)
14	astragalus methoside	Kim et al. (1999)
15	(kaempferol-3-*O*-(6-*O*-acetyl)-1,3-D-glucoside	Kim et al. (1999)
15	(quercetin-3-*O*-(6″-*O*- acetyl)-*β*-D-glucosyl-ranoside	Kim et al. (1999)
17	quercetin-3-*O*-*β*-D-glucoside	Kim et al. (1999)
18	kaempferol-3-*O*-*α*-L-rhamnopyranosyl-(1–6)-*β*-D-glucopyranoside	Kim et al. (1999)
19	quercetin-3-*O*-*α*-L-rhamnopyranosyl-(1–6)-*β*-D-ghcopyranoside	Kim et al. (1999)
20	quercetin-3-*O*-*β*-D-glucopyranosyl-(1–6)-*β*-D-glucopyranosi	Kim et al. (1999)
21	quercetin-3,7-2-*O*-*β*-D-glucopyranoside	Kim et al. (1999)
22	quercetin	Kim et al. (1999)
23	sangiferin A	Hao M. et al. (2021)
24	sangiferin B1	Hao Y. et al. (2021)
25	sangiferin B2	Hao M. et al. (2021)
26	kaempferol-3-*O*-*β*-D-glucopyranoside	Hao Y. et al. (2021)
27	kaempferol-7-*O*-β-*D*-glucopyranoside	Hao M. et al. (2021)
28	kaempferol-3,7-di-*O*-β-D-glucopyranoside glucopyranoside	Hao Y. et al. (2021)
29	quercetin-3-*O*-*β*-D-glucopyranoside	Hao M. et al. (2021)
30	morin	Hao Y. et al. (2021)
31	morin M-6,3′-di-*O*-*β*-D-glucopyranoside	Hao M. et al. (2021)
32	chalcopyranoside	Hao Y. et al. (2021)
33	morin chalcone b	Yang et al. (2010)
34	morin chalcone c	Yang et al. (2010)
35	3-*O*-rutinoside-quercetin	Thaipitakwong et al. (2018)
36	quercetin-3-*β*-D-glucopyranoside	Thaipitakwong et al. (2018)
37	astragaloside	Thaipitakwong et al. (2018)
		Hong et al. (2013)
38	Kaempferol-3-*β*-D-glucopyranoside	Thaipitakwong et al. (2018)
39	lignan-7-*O*-gentianoside	Araujo et al. (2015)
40	5,6 hydroxy lignan-7-*O*-rutinoside	Araujo et al. (2015)
41	quercetin-3-*O*-ribofuranosyl-2″-ribofuranosyl	Araujo et al. (2015)
42	quercetin-3-*β*-D-glucose	Kim and Jang (2011)
43	quercetin-3-*O*-glucose-6-acetate	Kim and Jang (2011)
44	kaempferol-3-*O*-rutinoside	Hong et al. (2013)
45	3-geranyl-3-isopreno-2,4,5,7-tetrahydroxyflavone	Niu et al. (2020)
	**Alkaloids**	Asano et al. (1994a)
46	1,N-methyl-1-deoxynogicin	Asano et al. (1994a)
47	2-*O*-*α*-D-galactosylamino-glycosyl-1deoxynogicin	Asano et al. (1994a)
48	fagomine	Asano et al. (1994a)
49	1,4-dideoxy-1,4-imino-D-arabinitol	Asano et al. (1994a)
50	1,4-dideoxy-1,4-imino-(2-*O*-*β*-D-glucopyranosyl)-Darabinitol	Asano et al. (1994a)
51	1α,2β,3α,4β-tetrahydroxy-demetropine	Asano et al. (1994a)
52	nortropanoline	Asano et al. (1994a)
53	D-glucopyranoside	Gryn-Rynko et al. (2016)
	**Polysaccharides**	
54	water-soluble polysaccharides (WSPSs/FMAP/MBBP-1/MBBP-2)	He et al. (2018)
55	hemicellulose A/B (HMCs-A/HCMs-c)	He et al. (2018)
56	pectin substances (PcSs)	He et al. (2018)
57	pectin polysaccharides (SDA/CMA-b1-1)	He et al. (2018)
58	acidic polysaccharide (Mp-3)	He et al. (2018)
69	acidic heteropolysaccharide (JS-MP-1)	He et al. (2018)
60	branched chain amyloid-like polysaccharide (CAM-a-1)	He et al. (2018)
61	MP1	He et al. (2018)
62	MP2	He et al. (2018)
	**Phenolic**	
63	benzofurans	Chan et al. (2020)
64	phenolic acids	Chan et al. (2020)
65	coumarins	Chan et al. (2020)
66	stilbene	Chan et al. (2020)
67	MoralsinA-Z	Naik et al. (2015)
68	neochlorogenic acid	Doi et al. (2001)
69	cryptochlorogenic acid	Robbins (2003)
70	chlorogenic acid	Robbins (2003)
71	caffeic acid	Zeni et al. (2017)
72	gallic acid	Zeni et al. (2017)

### 2.2 Alkaloid compounds

Since the 1990s, scientists have investigated the alkaloids found in mulberry trees’ leaves, fruits, and flowers. N Asano (Asano et al., 1994a; Asano et al., 1994b; Asano et al., 2001) et al. isolated and purified alkaloids from mulberry leaves and determined seven alkaloid compounds, namely, 1,N-methy-1-deoxynogicin, 2-*O*-*α*-D-galactosylamino-glycosyl-1deoxynogicin, fagomine, 1,4-dideoxy-1,4-mino-D-arabinitol, 1,4-dideoxy-1,4-imino-(2-*O*-*β*-D-glucopyranosyl)-Darabinitol, 1α,2β,3α,4β-tetrahydroxy-demetropine and nortropanoline. In these seven compounds 1-deoxynojirimycin (DNJ), golden buckwheat alkaloids and 2-O-α-D-galactopyranosyl-1-deoxynojirimycin (GAL-DNJ) these three alkaloids account for more than 80% of the alkaloids in mulberry leaves, and later studies have been conducted around these three compounds. In addition to the alkaloids mentioned above, mulberry leaves also contain benzyl D-glucopyranoside (Gryn-Rynko et al., 2016). Researchers from China also found an interesting phenomenon in the study of alkaloids in mulberry leaves and discovered that the content of alkaloids in mulberry leaves varies seasonally. Using a combination of hydrophilic chromatography and tandem mass spectrometry (HILIC-MS/MS), the researchers measured different varieties of mulberry leaves and found that the maximum DNJ content occurred in June or July, the maximum FAG content occurred in the spring in April or May, and the maximum Gal-DNJ and Glu-FAG content occurred in September or October (Han et al., 2018).

### 2.3 Polysaccharide compounds

Researchers have studied the polysaccharides of Morus alba including leaves, fruits, root bark and shoots since the 1990s. Among them, water-soluble polysaccharides (WSPSs/FMAP/MBBP-1/MBBP-2), hemicellulose A/B (HMCs-A/HCMs-c), pectin substances (PcSs), pectin polysaccharides (SDA/CMA-b1-1), acidic polysaccharide (Mp-3), acidic heteropolysaccharide (JS-MP-1), branched chain amyloid-like polysaccharide (CAM-a-1), MP1, MP2 (He et al., 2018). Meanwhile, the monosaccharides that make up the polysaccharides of mulberry leaves are mainly composed of glucose, galactose, arabinose, fructose, xylose, rhamnose, glucuronic acid, galacturonic acid and, to a lesser extent, mannose and sorbose (Stocker et al., 2006; Katayama et al., 2008).

### 2.4 Phenolic compounds

Polyphenols are one of the richer components contained in mulberry leaves, and so far researchers have isolated more than 140 polyphenols from mulberry leaves, with flavonoids and flavonoid components accounting for a relatively high proportion of polyphenols; the remaining classes of polyphenols are benzofurans, phenolic acids, coumarins, stilbene (Chan et al., 2020). One of the benzofuran compounds is MoralsinA-Z (Naik et al., 2015). The phenolic acids in mulberry leaves are neochlorogenic acid, cryptochlorogenic acid, chlorogenic acid, caffeic acid and gallic acid (Doi et al., 2001; Robbins, 2003; Zeni et al., 2017); the coumarins contain mainly substances such as desmoglein in addition to Moralsin mentioned above, while resveratrol belongs to the stilbene substances in mulberry leaves (Bubols et al., 2013). The main compounds of mulberry leaves covered in the article are shown in Table 1.

## 3 Safety evaluation of mulberry leaves

As a medicine, the safety of mulberry leaves should be evaluated before they are used in the treatment of diabetes and whether they can cause damage to other tissues or organs. The two types of extracts were subjected to acute, chronic and genotoxicity tests on rats and mice. The mice in the toxicity test were slightly more damaged than those in the ethanolic extract (Marx et al., 2016; Oliveira et al., 2016; Figueredo et al., 2018; Li et al., 2018). After mixing the powdered mulberry leaves with water, diabetics can take it with a significant improvement in blood sugar and no major adverse effects, but may experience occasional bloating and diarrhoea (Thaipitakwong et al., 2020).

## 4 *In vitro* and *in vivo* evaluation of the hypoglycaemic effect of mulberry leaves

To evaluate the hypoglycaemic potential of mulberry leaves, mulberry leaf extracts and the bioactive components therein *in vitro* and *in vivo*, the researchers tested them in different animal models.

Mulberry leaf powder was mixed with normal chow and fed to db/db mice for 20 weeks. At weeks 10, 15 and 20, body weight, pancreatic β-cell area, glucose tolerance, pancreatic endoplasmic reticulum stress markers and pancreatic β-cell nuclear value-added tests were performed in comparison with the control group (Suthamwong et al., 2020). Researchers tested the hypoglycemia effect of mulberry leaf powder in Wistar rats. 30 Wistar rats were divided into five groups and fed mulberry leaf powder and normal chow for 6 weeks (Salemi et al., 2016). Through these experiments, it was found that adding a dose of powdered mulberry leaves to the daily diet was a good way to control blood sugar. Extracts of mulberry leaves were obtained under different polar solvents, and for these extracts researchers have also conducted studies on hypoglycaemia. Dionísio HADA Silva (Silva et al., 2021b) et al. extracted the crude ethanolic extract of mulberry leaves with hexane and chloroform to obtain the less polar components α-linolenic acid, daustan-3-ol and ethyl linolenic acid in hexane and daustan-5-en-3-ol, palmitic acid and α-linolenic acid in chloroform, and used these extracted compounds in streptozotocin-induced The results showed that only the hexane extract reduced both fasting and postprandial blood glucose, while the chloroform extract showed better results on lipid and liver function by reducing triglyceride, LDL, alanine aminotransferase, aspartate aminotransferase and alkaline phosphatase levels. The n-hexane extract of Morus alba can also maintain body weight, maintain liver glycogen levels, improve oxidative stress levels and restore superoxide dismutase activity in the body. In addition to this, the n-hexane extract can improve insulin sensitivity and reduce glucose absorption in the small intestine. The n-hexane component of Morus alba has also been demonstrated to lower blood glucose in mice. In a diabetic mouse model, the n-hexane extract of Morus alba was administered and found to delay the digestion and absorption of carbohydrates, reduce postprandial blood glucose values and inhibit the activity of α-glucosidase (Silva et al., 2021a). The extract was obtained by extracting mulberry leaves with ethanol and 48 rats were grouped and treated by gavage for 11 weeks, after which the islet β-cell function, insulin resistance index, fasting blood glucose, plasma insulin levels and the histomorphology of islets were measured. The results showed that the ethanolic extract of Morus alba could improve hyperglycaemia, insulin resistance, dyslipidaemia, improve islet function and reduce islet damage in rats (Ji et al., 2021). In addition to the solvent extracts mentioned above, the aqueous extract of Morus alba also showed good performance in the control of blood glucose. The hypoglycaemic effect of aqueous extract of Morus alba was tested in C57BL/6J mice as well as *in vitro*, where α-glucosidase activity and protein glycosylation were measured. The results showed that the aqueous extract of Morus alba had a high rate of α-glucosidase inhibition, reduced serum glucose levels, serum free fatty acids, tumour necrosis factor-α and glycated haemoglobin, and improved renal damage and microbiota in the gut (Han et al., 2020). Researchers have chosen to use dichloromethane followed by extraction in an aqueous extract of mulberry leaves found to be tested for *in vitro* anti-glucose in components soluble in dichloromethane, and it has been reported that adipose tissue is one of the important sites of postprandial glucose uptake (Watson and Pessin, 2007). Therefore, in this study, an aqueous extract of mulberry leaves was added to 3T3-L1 adipocytes in the dichloromethane soluble fraction and the glucose consumption was compared over a 24 h period and the dichloromethane soluble fraction was found to increase glucose consumption in 3T3-L1 adipocytes in a dose-dependent manner (Hunyadi et al., 2013). The above study shows that mulberry leaves have a significant effect on diabetes when used as a raw drug or after a simple extraction.

### 4.1 Hypoglycaemic evaluation of mulberry leaves flavonoids

Takuya Katsube (Katsube et al., 2010) investigated the effect of quercetin 3-(6-malonylglucoside), a flavonoid in mulberry leaves, on glucolipid metabolism in diabetic mice, using C57BL/6J mice grouped on a high-sugar, high-fat diet and 10 g of mulberry leaf powder and quercetin 3-(6-malonylglucoside). The physiological and biochemical parameters were measured and found to be significantly lower in blood glucose, free fatty acids and low density lipoproteins, and oxidative stress levels were significantly improved in mice fed flavonoids. Carolina Morais Araujo (Araujo et al., 2015) studied the flavonoid content of mulberry leaves and the hypoglycaemic effect of mulberry leaf extract. The results showed that the flavonoids in mulberry leaves reduced the superoxide dismutase (SOD)-peroxidase (CAT) ratio, decreased metalloproteinase-2 activity, increased blood insulin levels and reduced hyperglycaemia-induced diabetes. According to the above study, the flavonoid component of Morus alba extract could be found to improve oxidative stress and blood glucose levels in diabetic rats. In a study on the control of blood glucose by flavonoid components of mulberry leaves, researchers found that a new flavonoid compound was also more effective in improving insulin resistance and controlling blood glucose. Sheng-Li Niu(Niu et al., 2020) identified this new geranylated flavonoid and its structural analogues by wave spectroscopy, and by studying its modulation of protein phosphatase 1B (PTP1B) D The new flavonoid was found to increase glucose consumption by cells with insulin resistance, as well as on glucose consumption by HepG2. These findings provide important evidence for the use of mulberry leaf flavonoids to regulate blood glucose.

### 4.2 Hypoglycaemic evaluation of mulberry leaf alkaloids

The alkaloids contained in mulberry leaves such as 1-deoxywildicamycin (DNJ) are very strong inhibitors of intestinal α-glucosidase (Asano et al., 1994b). Yatsunami et al. (2008) determined the DNJ content of 276 Japanese mulberry leaves and investigated the (DNJ) inhibitory activity on α-glucosidase using high performance liquid chromatography. Hu et al. (2019) investigated the effect of DNJ on glucose metabolism in streptozotocin-induced diabetic mice, and measured the relevant indexes after 2 weeks of treatment with DNJ in the modeling results. Hu et al. (2017) conducted a study on urinary metabolism in diabetic mice in which DNJ was used to lower blood glucose. In this study, selected indicators related to type II diabetes mellitus (T2DM) such as glucose, insulin, triglycerides, total cholesterol, nitrogen, malondialdehyde and creatinine were significantly reduced after 4 weeks of treatment, and the data of these indicators became closer to those of normal mice as the treatment period was extended.

### 4.3 Evaluation of sugar reduction by mulberry leaves polysaccharides

The polysaccharides (MLP) in mulberry leaves also play a very important role in the improvement of blood sugar. Isolation and purification of polysaccharides from Morus alba and antidiabetic studies by Zhang et al. (2014a) In his study, 40 Wistar rats were fed a high-fat, high-sugar diet and induced to become diabetic with streptozotocin, then treated with purified 99.8% mulberry leaf polysaccharide for 5 weeks, followed by oral glucose tolerance testing and serum collection and histomorphological observation of the pancreas and liver. The results showed that fasting blood glucose (FBG), glycated serum protein (GSP), total serum cholesterol (TC) and triglyceride (TG) levels were significantly reduced, body weight, fasting insulin (FINS), C-peptide (C-P), liver glycogen and serum high-density lipoprotein cholesterol (HDLC) were increased, in addition to the significant effect of Mulberry leaf polysaccharide (MLP) on pancreatic β-cell regeneration and insulin secretion, and the reduction of liver It also reduced the accumulation of fat in the liver of diabetic rats. After the hypoglycaemic study of mulberry leaf polysaccharide (MLP) mentioned above, Zhang et al. (2014b) further purified mulberry leaf polysaccharide by selecting products with molecular weights less than 10,000 to elute on a chromatographic column and purify the product to mulberry leaf polysaccharide II (MLP II) after the process was completed. The results showed that the purified MLP II was able to inhibit islet cell apoptosis and improve insulin secretion. The results showed that the purified MLPⅡ could inhibit the apoptosis of pancreatic β-cells and significantly improve the insulin secretion ability of pancreatic β-cells in diabetic rats. The role of MLP II in regulating blood glucose was further explored in terms of hepatic glucose metabolism and insulin signaling in diabetic rats by Ren et al. (2015) Oral glucose tolerance tests, liver glycogen content, glucose synthase (GS) activity and expression patterns of proteins and genes related to insulin resistance and insulin signalling, as well as biomarkers of oxidative stress and antioxidant enzyme activity were analysed in rats at the end of treatment with MLPII. The results showed that MLPII treatment significantly improved glucose tolerance, restored glycogen levels and GS activity in diabetic rats, and significantly improved insulin resistance in diabetic rats, in addition to significantly increased expression levels of insulin receptor substrate 2 (IRS2), phosphatidylinositol 3-kinase (PI3K) and protein kinase B (PKB/AKT), which are involved in insulin signalling, and protein tyrosine phosphatase 1B (PTP1B). The levels of 8-hydroxy-2-deoxyguanosine (8-OHdG) and malondialdehyde (MDA) in the liver of MLPII-treated rats were significantly reduced, and the activities of superoxide dismutase (SOD), catalase (CAT) and glutathione peroxidase (GPx) were significantly increased. From the above studies on the regulation of blood glucose by Morus alba polysaccharides, it can be seen that Morus alba polysaccharides have a significant effect on the maintenance of blood glucose homeostasis.

### 4.4 Hypoglycaemic evaluation of polyphenolic substances in mulberry leaves

Li et al. (2017) used UHPLC-HR-ESI-TOF-MS/MS to explore the antidiabetic polyphenols in mulberry leaves. The phenolic compounds were identified and quantified by UHPLC-HR-ESI-TOF-MS/MS. The antioxidant activity and antidiabetic effects of the phenolic compounds, including pheophytin 3-glucoside-5-glucoside and anthocyanin 3-glucosidic dimer, were analyzed *in vitro* using HepG2 and pancreatic β-cell line RIN-m5F. The results showed that compounds such as butyric acid and gallic acid glucoside had the greatest inhibitory effect on α-glucosidase activity, quercetin and cyanogenic glycosides had better antioxidant effects on cells, while dihydroquercetin and 1,5-dicaffeoylquinic acid were the main components protecting RIN-m5F cells from glucotoxic effects after rutin. Li et al. (2020) investigated that mulberry leaf polyphenols reduce postprandial glucose uptake by inhibiting the disaccharidase activity and glucose transport pathway in Caco-2 cells. The purification of mulberry leaf polyphenols identified its main components chlorogenic acid, benzoic acid and auriculoside and cultured mouse-derived Caco-2 cells in disaccharidase medium with sucrase as well as maltase solution, and by Varghese and Thomas. (2019) investigated the effect of polyphenols in mulberry leaves on the maintenance of healthy blood glucose levels in diabetic rats, and used HPLC to isolate and identify the polyphenolic components of mulberry leaves and fed a high-fat diet with streptozotocin. The study used HPLC to isolate and identify the polyphenolic components of mulberry leaves and fed a high-fat diet to induce a diabetic model in Wistar rats. After 4 weeks of treatment, it was found that mulberry leaf polyphenols significantly reduced blood glucose levels, decreased serum urea, creatinine levels, serum triglyceride and total cholesterol levels, and increased serum HDL-cholesterol levels.

## 5 Clinical trials of mulberry leaves in the treatment of diabetes mellitus

Based on the above it is known that the hypoglycaemic potential of mulberry leaves, mulberry leaf extracts and individual components has been well established in animals and has a very good effect in controlling blood glucose. However, the ultimate aim of mulberry leaves as a substance with a good hypoglycaemic effect is to improve the blood glucose levels and enhance the quality of life of diabetic patients. The results of these clinical trials will serve as direct evidence of the hypoglycaemic effect of mulberry leaf. The results of these clinical trials will serve as direct evidence of the hypoglycaemic effect of mulberry leaves. At present, clinical trials on the hypoglycaemic effect of mulberry leaves are based on the alkaloid 1-DNJ, which is extracted, isolated and purified from mulberry leaves, and can be used to demonstrate the hypoglycaemic effect of mulberry leaves.

In a study from Korea on the improvement of postprandial glucose response in pre-diabetic subjects with Mulberry leaf extract, researchers conducted a 4-week treatment with Mulberry leaf aqueous extract (MLAE) supplementation (5 g/day, containing 1-DNJ 3.6 mg/g) and placebo on postprandial glucose in 36 subjects with impaired fasting glucose (IFG) tolerance. Carbohydrate supplementation was administered to both groups at the end of the treatment period during the same time period, followed by measurement of serum glucose, insulin and C-peptide levels in both groups after 30 and 60 min. The results showed an attenuated postprandial glycaemic response in the MLAE group, especially at 30 and 60 min after loading (*p* = 0.003 and 0.0325 for glucose, *p* = 0.0005 and 0.0350, and C-peptide *p* = 0.0151 and 0.0864). In addition, the MLAE group had a significantly lower incremental area under the insulin curve than the placebo group (*p* = .0207) (Kim et al., 2015), The results of this clinical trial suggest that mulberry leaves can reduce postprandial blood glucose levels in pre-diabetic patients, leading to better regulation of blood glucose. In another clinical trial on mulberry leaves from Thailand, researchers treated diabetic patients with 1-DNJ from mulberry leaves separately from placebo, and after 12 weeks of treatment, the results of this clinical trial showed that mulberry leaves lowered postprandial blood glucose levels in pre-diabetic patients, resulting in better blood glucose regulation. Indicators found that Mulberry Leaf reduced fasting blood glucose (FPG) by 86.5 ± 99.0 mg/dL (*p* = 002.1) and glycated haemoglobin (HbA0c) by 11.0% ± 22.0% (*p* = 011.0) compared to baseline at the start of the trial, suggesting that Mulberry Leaf has a significant contribution to blood glucose improvement (Thaipitakwong et al., 2020). At the same time, no serious adverse effects were observed in either of these clinical trials after administration of Morus alba or 1-DNJ.

## 6 Epigenetics, diabetes mellitus and the link between mulberry leaves

Epigenetics is defined as heritable changes in gene function that occur without changes in DNA sequence, a characteristic that suggests that epigenetics is heritable. At the same time, the relationship between epigenetics and the metabolic diseases in which it is involved, and the study of the relationship between the two, is still relatively new and has attracted the attention of many researchers and scholars, with the development of diabetes and its own genetic changes becoming one of the hot topics of research (Ling and Rönn, 2019).

### 6.1 Epigenetic factors in type I diabetes mellitus

The mechanisms by which epigenesis occurs include DNA methylation, various non-coding RNAs and enzyme-catalyzed post-translational modifications of histones (Jiménez-Chillarón et al., 2012). There are many reasons for the genetic susceptibility to type 1 diabetes and its increased incidence, including epigenetic factors such as DNA methylation and histone modification of genes related to glucose metabolism, as well as external factors including environmental factors, lifestyle, social factors, infections and changes in the gut microbiome (Boerner and Sarvetnick, 2011; Lam and LeRoith, 2012). Among these are the close relationship between epigenetic factors and the organs of the liver, which control the patient’s own glucose metabolism, and the organs of the pancreas, which secrete insulin, where alterations in the epigenome of these organs can affect the development and progression of type 1 diabetes (MacFarlane et al., 2009; Stankov et al., 2013).

### 6.2 Epigenetic factors in typeⅡdiabetes mellitus

Researchers first studied the epigenetics of type II diabetes mellitus a decade ago (Barrès et al., 2009). Because the field of epigenetic research in type II diabetes is relatively new, with relatively few homogeneous target organs obtained, and because of the technical limitations of analyzing epigenetic changes on a genome-wide scale, more researchers are currently focusing on DNA methylation (Toperoff et al., 2012). Meanwhile another case-control study revealed that CpG loci in ABCG1, PHOSPHO1, SOCS3 and SREBF1 are strongly associated with the development of type II diabetes (Minn et al., 2005; Parikh et al., 2007). In addition to changes in methylation found in peripheral blood, alterations in DNA methylation have also been found in tissues or organs associated with glucose metabolism. In genetic testing of the islets of type II diabetic patients, researchers have found that deficient insulin secretion is associated with increased DNA methylation in pancreatic cells, and other studies have shown that higher blood glucose and glycosylated haemoglobin (HbA1c) levels in patients increase DNA methylation of related genes (Yang et al., 2011; Hall et al., 2018). CDKN1A, PDE7B and SEPT9 are among the genes with reduced DNA methylation in type II diabetic mice, and studies of CDKN1A and PDE7B have shown that promoter methylation negatively affects the transcriptional activity of these genes, leading to reduced insulin secretion when these three genes are overexpressed in pancreatic β-cells. In addition, overexpression of CDKN1A resulted in reduced insulin secretion and reduced islet beta cell value added. TCF7L2 (Dayeh et al., 2014).

An important feature of epigenetic change is that it is reversible and modifiable, a property that makes it a possible therapeutic target for disease (Kwak and Park, 2016). It has been found that this DNA methylation inhibitor has been widely used in metabolic diseases such as cancer, asthma and diabetes (Boyanapalli and Kong, 2015). In a study of curcumin’s ability to regulate blood glucose by inhibiting DNA methylation, researchers found that curcumin can reduce inflammation and oxidative stress in diabetic patients by reducing IL-1β, TNF-α and ROS levels in the body, which can lead to damage to pancreatic β-cells and reduced insulin secretion, resulting in increased blood glucose levels in patients (Tang et al., 2022). Similar to curcumin, the active substance in mulberry leaf can regulate blood glucose by regulating the levels of oxidative stress and inflammatory factors in the body, thereby protecting pancreatic islet cells, a result similar to that of curcumin. However, there are no international studies on the role of the active ingredients in mulberry leaves in treating diabetes by improving the epigenetics of the genes of diabetic patients and the genetic reasons for mulberry leaves regulating the levels of oxidative stress and inflammatory factors in the body to improve blood glucose are improved DNA methylation. The study of curcumin could provide a hypothesis on how mulberry leaf affects the epigenetics of diabetic patients by altering the methylation of DNA and thus treating diabetes, thus providing new ideas and avenues for mulberry leaf and even other herbs with good hypoglycaemic effects to treat the onset and development of diabetes through the epigenetics of diabetes.

## 7 Molecular mechanism of hypoglycemia in mulberry leaves

### 7.1 Mulberry leaf can improve oxidative stress to regulate blood sugar

**FIGURE 1:**
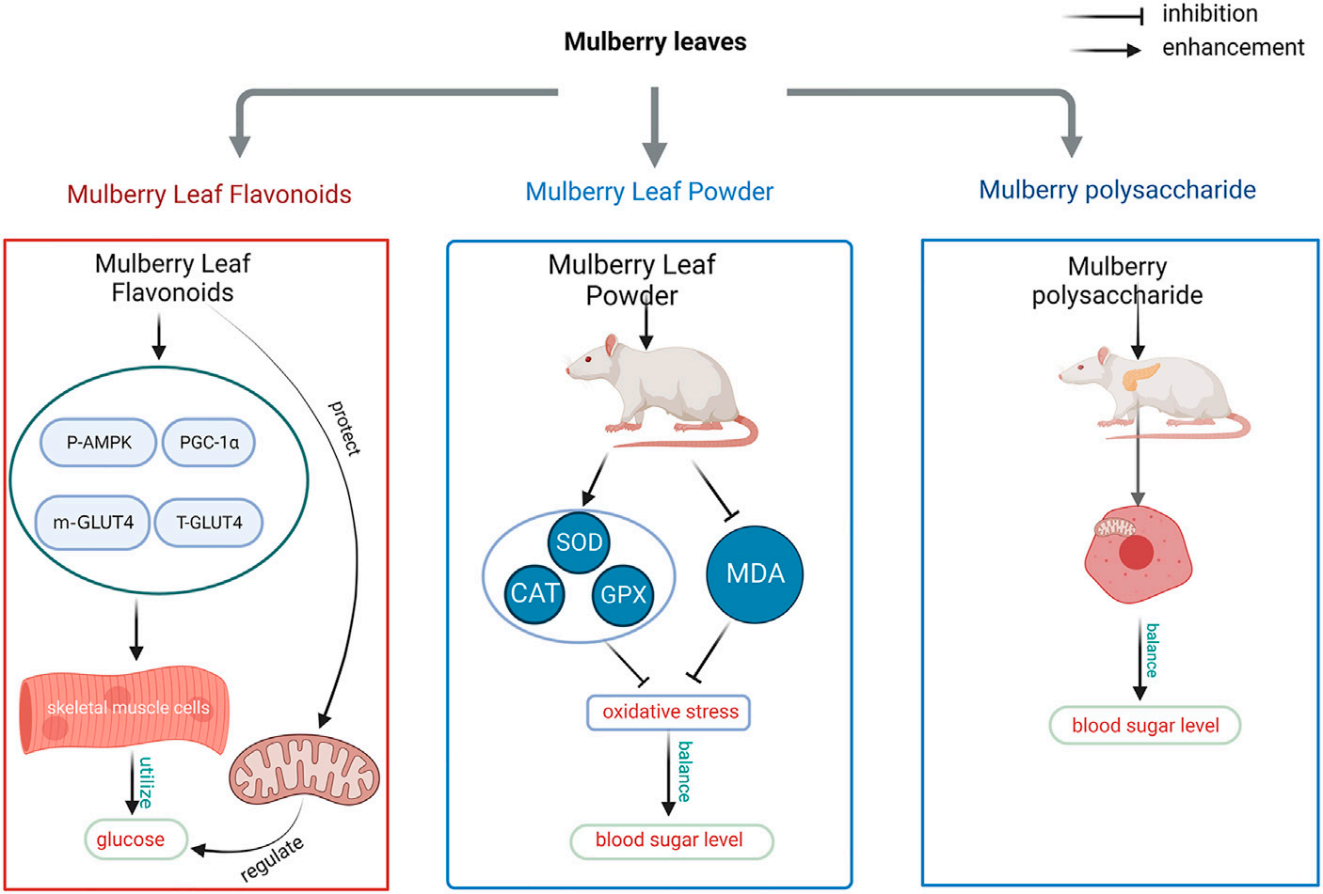
Mechanism of blood glucose regulation by improving oxidative stress in mulberry leaves.

Stress occurs when the level of oxidants in cells and blood exceeds the level of antioxidants produced by the body, and there is a strong link between oxidative stress and insulin resistance, which occurs in skeletal muscle tissue and in the development of type II diabetes (Henriksen et al., 2011). As research into the relationship between oxidative stress and diabetes has become more advanced, researchers have found that reactive oxygen radicals (ROS) and oxidative stress are increasingly responsible for the development of diabetes, and that the chemical composition of most cells in the body is altered by the high activity of ROS. The accumulation of lipid peroxides is the main cause of oxidative stress in the body, which in turn has an impact on blood glucose (Rehman and Akash, 2017). When lipid peroxides are high in the cells of skeletal muscle, ROS interact with insulin receptors and their downstream signalling pathways to affect islet function and cause islet cell dysfunction (Newsholme et al., 2014; Verdile et al., 2015). Researchers have experimentally found that the main sources of ROS production are electron transfer into oxygen molecules through respiratory metabolism in mitochondria to form superoxide anions (O2-) and ROS itself, which can also be activated by NADPH oxidase (Valko et al., 2007; Nowotny et al., 2015). The production of superoxide anions is accelerated by the production of nitric oxide (NO) in the body, and when the body’s endothelium is dysfunctional superoxide anion production neutralises NO production through the oxidation of NO to produce peroxynitrite, a pathway that initiates ROS and generates a cascade reaction that further exacerbates the increased oxidation of proteins, carbohydrates and fats. The metabolism of unsaturated fatty acids in the body also results in the oxidation of various lipid peroxides, such as 4-hydroxy-nonenal (HNE), 4-oxy-2-nonenal (ONE) and malondialdehyde (MDA) (Hopps et al., 2010). Studies have shown that flavonoids in mulberry leaves accelerate glucose uptake and utilization in skeletal muscle cells by increasing AMPK phosphorylation, increasing adenosine monophosphate-activated protein kinase-peroxisome proliferator-activated receptor gamma coactivator 1-α (PGC-1α) levels, and upregulating m-GLUT4H and T-GLUT4 protein levels, while also improving mitochondrial function to regulate blood glucose, The total flavonoids extracted from mulberry leaves were diluted at 5, 10, 20, 40 and 80 μg/mL to measure the cell viability and the above mentioned parameters in skeletal muscle cells (L6 cells), The results showed that the five concentrations of total flavonoids of Mulberry leaves did not have any significant effect on the cellular value-added capacity of L6 cells. The final concentration of 10 μg/mL of total flavonoids of Mulberry leaves was selected for *in vitro* experiments, which explained its improvement of blood glucose by the above molecular mechanism (Meng et al., 2020b). Insulin stimulates intracellular GLUT4 vesicles to increase GLUT4 in the cell membrane through cytosolic transport, whereas skeletal muscle cells are responsible for 80% of insulin-stimulated glucose uptake and glucose uptake and utilisation is mainly accomplished through glucose transport protein 4 (GLUT4), and enhanced PGC-1α enhances mitochondrial function and GLUT4 transport to the cell membrane function of the mitochondria (Leick et al., 2010; Hesselink et al., 2016; Klip et al., 2019). In their study of oxidative stress causing diabetes, researchers found that the body’s antioxidant system acts as a scavenger for ROS. The antioxidant system includes different types of functional components, which are divided into first and second line defence systems. The first line of defence includes preventive antioxidants using burst O2- and catabolism of H2O2 to exert antioxidant effects belonging to this class of oxidants are mainly some Enzymes such as superoxide dismutase (SOD), catalase (CAT), glutathione peroxidase (GPX) and glutathione reductase, as well as non-enzymatic molecules such as minerals. The second defence system of antioxidants has among others vitamin C, carotene and vitamin E (mainly α-tocopherol). The flavonoid component of mulberry leaf extract and mulberry leaf powder were used to treat male Wistar diabetic rats induced with streptozotocin (STZ) by measuring glucose 6-phosphate dehydrogenase (G6PDH), GPX, glutathione-S-transferase (GST), superoxide dismutase and catalase (CAT) as well as serum vitamin C and vitamin E levels and found that the flavonoid component of mulberry leaf The phytochemicals contained in mulberry leaf powder such as vitamin C and carotene can regulate oxidative stress by reducing lipid oxidation, while the latter is a precursor to vitamin A and can also improve oxidative stress (Garg and Bansal, 2000; Ng et al., 2000; Andallu et al., 2012) The researchers studied the improvement of oxidative stress in the pancreatic β-cells of diabetic rats by measuring the activities of mitochondrial cytochrome C oxidase (CCO), succinate dehydrogenase (SDH) and superoxide dismutase (SOD) as well as malondialdehyde in the pancreatic β-cells of rats. The treatment was carried out for 8 weeks (once daily) using 100 mg/kg of Morus alba at a concentration of 6 mg/mL, By measuring blood glucose values and using spectrophotometric methods, it was found that the blood glucose levels were significantly reduced, CCO and SDH values were significantly restored, SOD activity was reduced and serum malondialdehyde levels were increased in comparison with the model diabetic rats (Figure 1) (Liu et al., 2017).

### 7.2 Mulberry leaves can improve insulin resistance to regulate blood sugar

**FIGURE 2:**
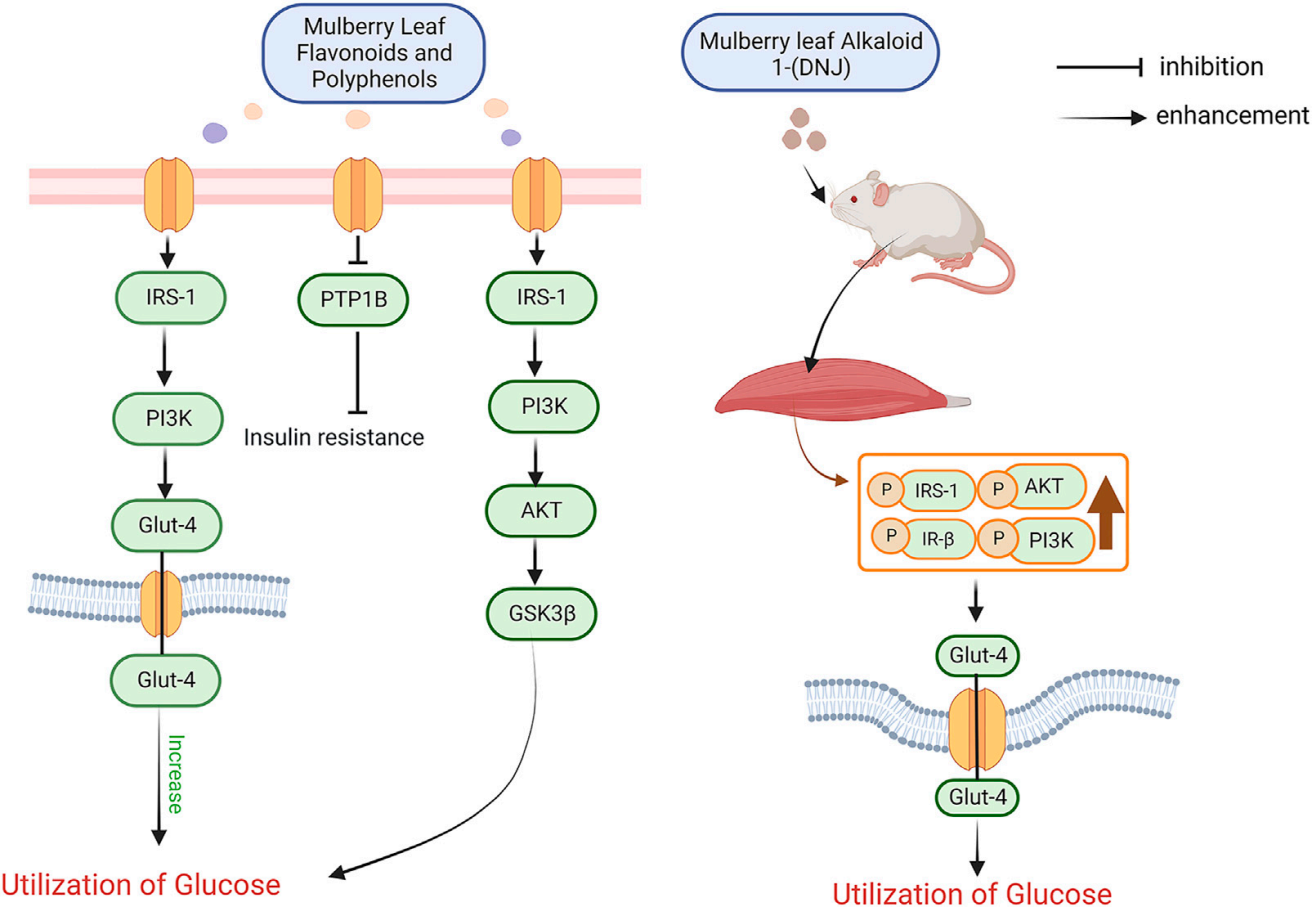
Mechanisms by which mulberry leaves improve insulin resistance to regulate blood glucose.

After the first discovery of insulin by researchers in 1922, it was widely believed that the cause of diabetes was entirely due to insufficient insulin secretion. 10 years later, researchers studied the function of the pancreas in diabetic patients and suggested that the cause of diabetes was not solely due to insufficient insulin secretion but also a large part of insulin insensitivity. Later scientists used a series of methods such as radioimmunoassay to prove the claim that lack of insulin sensitivity produces diabetes and proposed the basis of the corresponding mechanism (Yalow and Berson, 1960; Olefsky et al., 1973; Kahn et al., 1976; Kolterman et al., 1980; Himsworth, 2013). The potential mechanisms of insulin resistance are diverse and include cellular inflammation, endoplasmic reticulum stress and other alterations in the function of the body’s cells (Houstis et al., 2006; Blüher, 2016). At the same time, key substances and signalling pathways affecting insulin resistance have been identified, such as insulin receptor substrate (IRS), tumour necrosis factor-α (TNF-α), interleukin-6 (IL-6), protein tyrosine phosphatase 1B (PTP1B), PI3K-Akt, ERK, and adipocytokines (Hosogai et al., 2007; Ozaki et al., 2016; Zhang et al., 2019). When food intake is inadequate in the daily diet, the liver produces glucose and transports this glucose to the blood and tissues to maintain the body’s blood glucose stability and normal physiological activity of the tissues, a process that relies on fatty acids and glycerol from fat (Rizza, 2010). When sufficient food is consumed, pancreatic β-cells begin to secrete insulin to promote the body’s metabolism, and in the metabolism of glucose insulin promotes the synthesis of glycogen in skeletal muscle and adipose tissue, which are glucose-consuming tissues (Moore et al., 1991). Insulin also controls the central nervous system to reduce the secretion of glucagon, which raises blood glucose, and to reduce appetite (Asplin et al., 1981; Greenbaum et al., 1991). The specific mechanism by which insulin produces its effects is as follows. In cells, the function of insulin is mainly influenced by the insulin receptor tyrosine kinase (IRTK), which, when in the outer region of the tyrosine kinase protein, causes a conformational change in the tyrosine kinase and a shift in its residues towards phosphorylation, resulting in the complete activation of phosphotyrosine-binding proteins such as insulin receptor substrate (IRS), growth factor receptor binding protein-2 and growth factor receptor binding protein-2. protein-2 and growth factor receptor binding protein-10, and a range of initiator proteins and receptor proteins bound to them (Youngren, 2007). The phosphorylated IRS activates phosphatidylinositol-3-hydroxyl kinase (PI3K) and synthesizes phosphatidylinositol-4,5-bisphosphate (PIP2) into phosphatidylinositol-3,4,5-trisphosphate (PIP3), which is then attached to the lipid membrane of PIP3, allowing Akt to be phosphorylated by PDK1. The phosphorylated Akt signals downstream to skeletal muscle, adipose, liver and other tissues to promote glucose utilization by these tissues (Khalid et al., 2021). In skeletal muscle insulin moves and fuses glucose transporter protein 4 (GLUT4) to the plasma membrane of skeletal muscle cells, activates the AS160 protein that controls the encapsulation of GLUT4 protein vesicles and acts as a transporter, induces GLUT4 displacement through the RAC1 protein that promotes the induction of cortical actin reorganization by Akt, and increases glycogen synthase 3 (GSK3) through the Akt pathway phosphorylation and activation of protein phosphatase 1 (PP1) activity to promote increased glycogen synthase (Gys) and decreased phosphorylation of Gys (Cross et al., 1995; Newgard et al., 2000; Chiu et al., 2011; Leto and Saltiel, 2012). In hepatocytes insulin reduces hepatic gluconeogenesis by inhibiting Akt-induced phosphorylation of forkhead box O1 (FOXO1), thereby reducing and preventing transcription and activation of gluconeogenic gene expression such as glucose-6-phosphatase (G6PC) and phosphoenolpyruvate carboxylase (PEPCK) (Dong et al., 2008; Tzivion et al., 2011). At the same time insulin also increases hepatic glycogen synthesis by regulating Gys (GYS2 in the liver) like skeletal muscle cells and by regulating glycogen phosphorylases *via* GSK3 and PP1. In summary insulin resistance is defined as the inability of certain tissues in the body to receive feedback to normal insulin levels in the body and the need for higher insulin levels to ensure that the body produces a normal response to insulin. Whereas skeletal muscle and liver are the main sites of insulin signalling, these tissues can be understood as key to the occurrence of insulin resistance. Researchers evaluated mulberry leaf extracts for improving insulin levels and lowering blood sugar, and found that mulberry leaf substances with hypoglycaemic effects such as mulberry leaf flavonoids, mulberry leaf polyphenols, mulberry leaf alkaloids and mulberry leaf polysaccharides all have therapeutic effects on reducing insulin resistance. The researchers used mulberry leaf flavonoids and polyphenols at a dose of 2 g/kg according to the body weight of the rats for a 4-week intervention (once daily) in streptozotocin-induced diabetic rats and found that mulberry leaf flavonoids and polyphenols were effective in reducing insulin resistance in diabetic rats at the end of the intervention. The researchers then investigated the hypoglycaemic mechanism of these two substances and found that they could effectively improve insulin resistance and lower blood glucose through the IRS-1/PI3K/Glut-4 signalling pathway, and that the binding of the insulin receptor (IR) to its corresponding substrates could also affect the activation of one of these signalling mechanisms in type II diabetes. One of the signalling pathways activated is the adenosine 50-monophosphate-activated protein kinase and phosphatidylinositol 3-kinase (PI3K), enzymes that over-regulate glycogen synthase and thus control glycogen metabolism and storage. When PI3K is activated, glucose transporter protein 4 (GLUT-4) is translocated to the cell membrane, thereby controlling glucose uptake in the target tissue. Researchers have measured the levels of IRS-1, PI3K p85a and GLUT-4 mRNA and corresponding proteins in this signalling pathway and found that activation of the IRS-1/PI3K/Glut-4 signalling pathway reduced insulin resistance and thus improved blood glucose levels (Zisman et al., 2000; Ooms et al., 2009; Lee et al., 2012; Cai et al., 2016). Recently, a new class of flavonoids was extracted from mulberry leaves that inhibited protein tyrosine phosphatase 1B (PTP1B), which was found to be effective in improving insulin resistance glucose maintenance. PTP1B is an important factor in signal transduction and plays an important role in protein phosphorylation, activating PI3K, which activates PDK and Akt/PKB, the downstream signals of PI3K. IRS1-PI3K-Akt-GSK3β signalling pathway to promote glucose uptake and improve insulin resistance (Paudel et al., 2018; Tian et al., 2019; Meng et al., 2020a; Niu et al., 2020; Yang et al., 2020). The alkaloid component 1-deoxywildicin 1-(DNJ) in mulberry leaves is also very important in improving insulin resistance for glycaemic control. The alkaloid 1-(DNJ) extracted from mulberry leaves is currently known as a good inhibitor of alpha-glucosidase for the treatment of diabetes, and it also has the ability to improve insulin resistance in this way to synergistically control diabetes. The researchers used 20, 40 and 80 mg/kg of 1-DNJ to intervene in diabetic mice by intravenous injection for 4 weeks. The results showed that 1-DNJ improved insulin resistance, increased insulin sensitivity and lowered blood glucose, all in a dose-dependent manner. Molecular studies have shown that 1-DNJ increases the phosphorylation of Akt, PI3K, IRS1 and IR-β in skeletal muscle and finally leads to an increase in membrane displacement of GLUT4, making it possible for 1-DNJ in mulberry leaves to improve insulin resistance and reduce blood glucose (Liu et al., 2015). The mechanism by which mulberry leaf polysaccharides improve insulin resistance with is similar to that of the new flavonoids mentioned above, which also activates the PI3K/Akt pathway by inhibiting the expression of PTP1B to achieve improved insulin resistance and lowered blood glucose (Figure 2) (Ren et al., 2015).

### 7.3 Mulberry leaves can control blood sugar by inhibiting the absorption of disaccharides

One of the typical features of diabetes is an abnormally high blood glucose level, which is diagnosed in diabetic patients after a prolonged fast of more than 126 mg/dL or more than 7 mmol/L. In non-diabetic patients, blood glucose is usually maintained at 70–100 mg/dL or 3.9–5.5 mmol/L 1 hour after eating, and most of them return to pre-meal status within 2–3 h The majority of patients return to their pre-meal state within 2–3 h (American Diabetes Association, 2001) Diabetes can also be diagnosed when blood glucose exceeds 200 mg/dL or 11 mmol/L measured 2 h after a meal. When the body is in a state of postprandial hyperglycaemia, the pancreatic β-cells are stimulated to gradually decrease their insulin secretion levels, increase oxidative stress in the pancreatic β-cells, and increase the rate of apoptosis in the pancreatic β-cells, which results in the progressive conversion of non-diabetic patients to diabetic patients over time (Poitout and Robertson, 2002; Brownlee, 2003; Kaiser et al., 2003). Elevated postprandial hyperglycaemia not only increases the risk of diabetes but also increases the risk of cardiovascular disease (Gerich, 2006).

Inhibition of α-glucosidase and α-amylase activities, which are involved in carbohydrate digestion, can effectively reduce the rise in postprandial hyperglycemia. α-glucosidase inhibition reduces the non-reducing terminal hydrolysis of oligosaccharides in food after eating, thereby delaying the digestion of carbohydrates in the small intestine and the absorption of glucose in the small intestine, a mechanism that plays a very important role in reducing postprandial hyperglycemia. This mechanism plays an important role in reducing postprandial hyperglycaemia (Kumar et al., 2011). Similarly inhibition of alpha-amylase activity slows down the conversion of ingested starch into glucose in the human small intestine, and by this mechanism can also reduce postprandial blood glucose levels (Pang et al., 2021) The active ingredients in mulberry leaves can control blood glucose by inhibiting the activity of both glycolytic enzymes. The most well-known and representative active substance among the components of mulberry leaves with dual enzyme inhibition activity is the mulberry leaf alkaloid 1-Deoxywildicamycin (1-DNJ). The mechanism by which 1-DNJ reduces the activity of α-glucosidase in the intestine may be that 1-DNJ binds to part of the catalytic structure of α-glucosidase and occupies its active site through hydrophobic interaction, inhibiting α-glucosidase activity in a competitive and reversible manner, In this study, 1-DNJ was compared with the hypoglycemic agent acarbose in terms of its α-glucosidase inhibitory activity, and the IC50 values were 35.62 ± 1.40 μM and 60.75 ± 1.90 μM respectively (He and Lu, 2013; Wu et al., 2018). Researchers have also found that the enzyme inhibitory activity of 1-DNJ is even comparable to that of clinically known α-glucosidase inhibitors such as acarbose, voglibose and miglitol. By gavage treatment (once daily) of diabetic rats induced with streptozotocin (STZ) in combination with a high-fat diet with 20 mg/kg of 1-DNJ according to the body weight of the rats in the positive group and 20 mg/kg of acarbose in the positive group, it was found that within 9 days of administration DNJ improved blood glucose more than the positive acarbose group and that it took longer for blood glucose to return to pre-treatment levels after treatment was stopped in the DNJ group than in the acarbose group (Miyahara et al., 2004; Kong et al., 2008; Kuriyama et al., 2008; Takasu et al., 2018). From the above studies on blood glucose regulation in animal models of diabetes, researchers found that DNJ inhibited the amount of glucose transport proteins in the intestine, such as SGLT1 in the brush border membrane of the small intestine and Na+/K + -ATP and GLUT2 in the basolateral membrane of the small intestine (Li et al., 2013a; Li et al., 2013b). The flavonoids in mulberry leaves also have good inhibitory effects on α-glucosidase. Researchers used column chromatography to elute and separate the aqueous extract of mulberry leaves to obtain flavonoids including rutin, isoquercitrin, kaempferol-3-0-rutinoside and astragaloside, and used these compounds to conduct *in vitro* α-glucosidase inhibition experiments using microplate method, and found that these compounds The inhibition rates of these compounds were found to be dose-dependent for α-glucosidase,Their IC_50_ values were 13.19 ± 1.10, 116.7 ± 1.17, 365.4 ± 1.05 and 15.82 ± 1.11 μM respectively (Hong et al., 2013). In addition to the inhibition of α-glucosidase by extraction of the aqueous fraction, the flavonoids obtained from the extracted fraction of mulberry leaves with non-polar components were administered to diabetic mice, and after a period of treatment, the oral carbohydrate tolerance and blood glucose values of the diabetic mice were improved, and the activities of α-amylase and α-glucosidase were inhibited (Silva et al., 2021a). It has also been shown that the addition of flavonoid components (5,6,7-trihydroxyflavone sapogenins) to 1-DNJ-containing diets can have a synergistic effect by increasing the inhibition of α-glucosidase by 1-DNJ, probably due to the fact that flavonoid sapogenins can act as an inhibitor that can bind to the non-competitive position of maltosidase-glucoamylase, allowing 1-DNJ to interact with α-glucoamylase. maltosidase-glucoamylase, thus exerting its effect of inhibiting α-glucosidase activity (Dong et al., 2020).

### 7.4 Mulberry leaves can control blood sugar by regulating lipid metabolism in the body

There is also an inextricable link between lipid metabolism and diabetes, with the number of overweight and obese patients consistently increasing over the last few decades in the US through military recruitment records, and evidence that 61% of the prevalence of diabetes can be attributed to obesity. One of the causes of insulin resistance is the level of blood fatty acids (FA) secreted by adipose tissue. In 1963 the scientist Randle suggested that blood fatty acids broken down in fat compete with glucose for metabolism, using a glucose clamp technique, dividing subjects into those injected with a dose of fatty acids and those injected with a dose of placebo, with the former showing significant muscle resistance to the use of glucose, and the latter showing none. This manifestation, which was specified as an increase in glycolysis and a decrease in glycogen formation, is one of the characteristics that we see in diabetic patients, and therefore fatty acid levels can be used as a will strong evidence of insulin resistance (Kelley et al., 1999; Kelley et al., 2001; Bloomgarden, 2003). Based on these studies, researchers examined the composition of the fatty acid profile in healthy people and diabetics in relation to the type of diabetes and found that both type I and type II diabetics showed abnormalities in triglycerides and cholesterol in fatty acids and reduced glycerides in sphingolipids, which are involved in apoptosis, immune function, inflammation and oxidative stress (Seigneur et al., 1994). Further experiments have found that increased fatty acids disrupt the balance of glucose metabolism in myocytes and have a lipotoxic effect on pancreatic β-cells, which also contributes to the development of diabetes (Keller, 2006). In addition to releasing free fatty acids, which affect blood glucose, fat also releases substances such as leptin, lipocalin, resistin, tumour necrosis factor-α and interleukin 6, which contribute to the development of insulin resistance. Leptin is a peptide-like hormone produced by adipose tissue and its concentration in the blood shows a positive correlation with insulin sensitivity (Hotamisligil et al., 1997; Iwata et al., 2001; Steppan et al., 2001; Yamauchi et al., 2001). Overall some of these factors secreted by adipocytes are beneficial in managing blood glucose levels but in the long term increased levels of adiposity will place a burden on those who control blood glucose.

Mulberry leaves can effectively address the above issues, both in terms of the total extract of mulberry leaves and the individual components thereof. The aqueous extract of Morus alba was used to study lipid metabolism in rats fed a high-sugar, high-fat diet. The effect of the aqueous extract of Morus alba on body weight, adipose tissue in the epididymis, serum biochemical parameters and genes/proteins related to lipid metabolism and lipid metabolism were investigated. The aqueous extract of Morus alba was found to be effective in improving body weight, reducing epididymal and hepatic fat accumulation, lowering serum triglycerides, LDL cholesterol, portal aminotransferase, alanine aminotransferase levels and elevating HDL cholesterol levels, upregulating genes related to lipid degradation such as PPARα, ACC, Fas and HSL, and improving lipid metabolism by improving the rats’ own n-3 polyunsaturated fatty acids. Phosphorylated AMPK increased the expression of PPARα, which regulates lipoprotein metabolism, inhibited the expression of ACC, a gene downstream of lipogenesis that catalyzes the production of malonyl coenzyme A to regulate fatty acid synthesis, and Fas, which promotes lipid synthesis, and elevated the expression of ADPN and its corresponding receptors AdipoR1 and AdipoR2. ADPN can bind to its receptors AdipoR1 and AdipoR2 to initiate AMPK phosphorylation, and these two receptors have levels of intracellular neuroendocannabinoids associated with insulin resistance, and ADPN can inhibit glucose production in the liver in addition to its effects on lipid metabolism, as confirmed by measurements of related genes or proteins (Yamauchi et al., 2001; Ahmadian et al., 2010; Chang et al., 2013; Fullerton et al., 2013; Fang and Judd, 2018). As the potential of mulberry leaves in lipid-lowering convenience was explored, researchers used network pharmacology to explore the active ingredients and key targets as well as *in vitro* validation using HepG2 cells to obtain the lipid-lowering mechanism of mulberry leaves. In this process, it was found that mulberry leaves may act through AKT, MAPK and IL-6 key targets, and by predicting that the flavonoid component is more effective in lowering lipids, the researchers extracted the flavonoid component and conducted *in vitro* validation. They found that in treating HepG2 cells with lipid aggregation, the flavonoid component of mulberry leaves could downregulate the secretion of inflammatory cytokines, inhibit lipid droplet formation, reduce TC, TG, HDLc and LDLc levels, and improved the expression of PI3K, Akt and Bclxl genes and proteins through the regulation of PI3K/Akt/Bclxl signalling pathway to exert hypolipidemic effects, The cytotoxicity of the mulberry leaf flavonoid composition showed no significant cytotoxicity at concentrations ranging from 12.5 to 200 μg/mL. In the present study, mulberry leaf flavonoid components were selected at concentrations of 25, 50 and 100 μg/mL and the results showed a dose-dependent form of improvement of the above indices by mulberry leaf flavonoid components (Li et al., 2021a). In contrast, polyphenols in mulberry leaves regulate lipids by improving HDL levels (Zeni et al., 2017). The polysaccharides in Morus alba inhibit the activity of phospholipase, an important enzyme associated with obesity prevention, to reduce fat absorption and affect lipid metabolism by reducing fat accumulation in the liver (Ben Bacha et al., 2018; Li et al., 2021b).

### 7.5 Mulberry leaves can treat diabetes by improving the microbial environment in the gut

The microbial system of the intestine in mammals is composed of four main phyla, namely, Actinobacteria, Bacteroidetes, Thick-walled Bacteria and Aspergillus, which play a crucial role in the regulation of metabolism and the body’s internal environment in mammals (Qin et al., 2010). The number of bacteria in the gut is approximately 100 trillion, which is three times the number of human cells (Bianconi et al., 2013). Based on the above, researchers have recently focused more and more on the gut microbial system as an “organ” with special functions, which can change when the body’s daily diet, lifestyle habits change, and when the body is attacked by diseases and other external influences, and these changes These changes can have new effects on the human body (Xu et al., 2007; Fukuda and Ohno, 2014). Researchers in China have analysed the gut microbes of type II diabetics and healthy humans using the LEfSe method of microbiological analysis and have shown significant differences in 43 gut microbes between the two (Wang et al., 2020). Microbes in the gut are involved in obesity, insulin resistance and chronic inflammation, all of which are more or less associated with the development of type II diabetes (Saad et al., 2016; Mouries et al., 2019; Lee et al., 2021). The reason for this may be that as the balance between the gut microbes and the body is disrupted, the permeability of the human gut is accelerated allowing substances produced by the gut microbes such as endotoxins (LPS) to enter the bloodstream and organs through the gut, and these substances stimulate the body’s immune cells, causing inflammation, oxidative stress and a range of other hazards, and as these substances are linked to some of the body’s signalling pathways regarding the development of diabetes. The progressive progression of the body towards diabetes is made possible by the combination of these substances with proteins in some of the signalling pathways involved in the development of diabetes (Cani et al., 2008).

Gut microbes act during the period from pre-diabetes to diabetes, a pathological state in which blood glucose levels have been above the threshold of normal blood glucose levels for some time, but have not yet reached a diagnosis of diabetes, which greatly increases the risk of developing diabetes (Tabák et al., 2012). The reduced abundance of *Clostridium perfringens* and Eosinophilus mucilaginosa and the lower abundance of metagenomic linkage groups (MLGs) from *C. perfringens* and *Proteus* vulgaris and MLGs from *Escherichia coli, Streptococcus* salivarius and *Proteus* euglena in pre-diabetic patients suggest a complex interaction between pre-diabetic patients and gut microbes (Allin et al., 2018; Zhong et al., 2019). By analysing the gut microbes of patients with diagnosed diabetes and healthy individuals, researchers have shown that the number of butyrate producing microbes is much higher in the gut than in type II diabetics, and that butyrate provides energy to the intestinal epithelium to maintain a healthy gut, reducing the permeability of the gut and reducing the overflow of inflammatory factors to reduce inflammation in the body. In addition, butyrate can increase the use of glucose by skeletal muscle cells and cardiac muscle cells, inhibit hepatic glycogen xenobiogenesis, enhance the breakdown of fat and improve the efficacy of insulin. The correlation between *Lactobacillus* species and glycated haemoglobin and fasting blood glucose levels is moderate (Lihn et al., 2005; Larsen et al., 2010; Karlsson et al., 2013; Umirah et al., 2021).

Researchers have also demonstrated in animal models that altered gut microbes can affect blood sugar. Researchers studied the microbial species in the gut of db/db mice with diabetes and used rRANA gene sequencing to find that 17 microbial species were altered in the gut of db/db mice, 10 of which showed an increasing trend and the remaining seven showed a decreasing trend. Transplanting faeces from diabetic mice as well as normal mice into germ-free mice reveals that the germ-free mice Significant changes in body weight, fasting blood glucose, urine and food intake, as well as in the composition of the gut microbes were found (Yu et al., 2019).

Mulberry leaf can be used to regulate blood glucose by improving gut microbes, both in terms of the overall composition and a single component of it. The aqueous extract of Morus alba was used to treat mice fed a high-fat, high-sugar diet and streptozotocin for 10 weeks. Daily dose of 250 mg/kg based on rat body weight (once daily). A comparison of the intestinal microbial counts in the treated and control groups showed that the aqueous extract of Morus alba reversed the abundance of Actinobacteria and Bacteroidetes and the ratio of bacteria to Bacteroidetes in the intestines of the experimental mice. This suggests that Mulberry leaf can effectively alleviate type II diabetes by regulating the host-microbe metabolic axis (Du et al., 2022). The ethanolic extract of Morus alba also played a significant role in reversing the microbial dysbiosis in the intestinal tract. In diabetic rats induced with high-fat diets and streptozotocin, after 5 weeks of administration of the ethanolic extract of Morus alba at a dose of 200 mg/kg of body weight (administered once daily), the abundance of microorganisms in the intestinal tract was found to be 1.06 times higher than in diabetic rats, while the abundance of The abundance of actinomycetes decreased by 41.6%, a level that was very close to that of normal rats. There was also a significant improvement in blood glucose values (Liu et al., 2021). The corresponding polysaccharide component of Mulberry leaf can also regulate blood glucose by improving microorganisms in the intestinal tract. The treatment of diabetic mice with Mulberry leaf polysaccharide was carried out for 19 weeks at a dose of 100 mg/kg in the high dose group and 100 mg/kg in the low dose group (once daily). infiltration of inflammatory factors and attenuated intestinal permeability. The results of the analysis of the microbial population in the intestinal tract showed that Mulberry leaf polysaccharide treatment group could reduce the abundance of *Lactobacillus*, Doberman, *Streptococcus*, Lactococcus and Thiobacillus, which were positively associated with insulin resistance, and increase the abundance of *Bacteroides, Haemophilus*, Ackermannia and Anaerobes, which were negatively associated with insulin resistance, and that Mulberry leaf polysaccharide could reduce fasting blood glucose, reduce insulin resistance and enhance insulin sensitivity, thus it could be concluded that Mulberry leaf polysaccharide could also regulate the number of intestinal microorganisms and their microbial balance to regulate blood glucose (Zhao et al., 2022). The researchers used diabetic mouse models treated with 125 mg/kg, 62.5 mg/kg and 31.25 mg/kg for 28 days (once daily) and found that 1-DNJ in mulberry leaves increased the production of *Lactobacillus* and Bifidobacterium, which inhibit pathogens and fight metabolic diseases, *Lactobacillus* and Bifidobacterium, which regulate insulin secretion and reversal of insulin resistance, and Wertheria, which reduce the secretion of pro-inflammatory factors, in diabetic mice. These findings link 1-DNJ, an alkaloid component of mulberry leaves, to intestinal microorganisms and blood glucose, among others, by increasing the number of *Bacillus* mimicus, which regulates insulin secretion and reverses insulin resistance, and reducing the secretion of pro-inflammatory factors (Hu et al., 2019).

Mulberry leaf is also very effective in treating complications caused by diabetes. The later stages of diabetes can cause damage or failure of different organs, such as to the kidneys, blood vessels, and the retina. Mulberry leaves are also effective in the treatment of diabetic complications. The active substances in mulberry leaves reduce the renal damage caused by diabetes and reduce inflammation and fibrosis in the kidney by modulating the activity of peroxisome proliferator-activated receptor γ (PPARγ) (Gurukar and Chilkunda, 2018). Mulberry leaf has also been shown to have better therapeutic effects in diabetic-induced vascular disease. Researchers have found that the antioxidant components of mulberry leaf extracts are absorbed through the intestinal tract and reach the bloodstream, increasing vascular elasticity and improving blood pressure levels in a rat model of diabetes by binding to cells in the vascular endothelium (Naowaboot et al., 2009). Similarly, the active substances in mulberry leaf also have a beneficial effect on diabetic retinopathy, as mulberry leaf extract scavenges free radicals in the body, reduces the expression of pro-inflammatory factors and oxidative stress markers in the retina, as well as downregulates the expression of caspase-3 and Bax and upregulates the expression of Bcl-2, while reducing the expression of vascular endothelial growth factor in the retina. Mulberry leaf has a protective effect on the retina of diabetic patients (Mahmoud et al., 2017). Overall, the alleviation of diabetic complications by mulberry leaves seems to be related to the improvement of oxidative stress in the body, the exact mechanism of which is not yet well understood, but it is undeniable that mulberry leaves have an important role in the field of combating diabetic complications. The molecular mechanisms involved in the treatment of diabetes with mulberry leaves covered in the article are shown in Table 2.

**TABLE 2:** Molecular mechanisms of mulberry leaves in the treatment of diabetes mellitus.

Treatment modalities	Experimental models	Molecular mechanisms	References
**Improving oxidative stress**	Diabetic rats	↑SOD, CAT, GPX, G6PDH	Garg and Bansal (2000)
			Ng et al. (2000)
		↓ MDA	Andallu et al. (2012)
			Liu et al. (2017)
	Islet β-cells	↑ CCO, SDH, SOD	Liu et al. (2017)
**Improving insulin resistance**	Diabetic rats	↑IRS-1/PI3K/Glut-4	Cai et al. (2016)
	HepG2-cells	↑IRS1/PI3K/Akt/GSK3β	Niu et al. (2020)
	Diabetic rats	↑IRS1/PI3K/Akt/GLUT4	Liu et al. (2015)
**Inhibits disaccharide absorption**	Diabetic rats	↓SGLT1, Na+/K + -ATP, GLUT2	Li et al. (2013a)
			Li et al. (2013b)
	Diabetic mouse	↓α-amylase, α-glucosidase	Silva et al. (2021a)
**Regulation of lipid metabolism**	Diabetic rats	↑PPARα, Fas, HSL	Yamauchi et al. (2001)
			Ahmadian et al. (2010)
		↑pAMPK, ADPN, AdipoR1, AdipoR2	Chang et al. (2013)
		↓ Hepatic glucose production	Fullerton et al. (2013)
			Fang and Judd (2018)
	HepG2-cells	↓TC, TG, HDLc, Inflammatory factor secretion	Li et al. (2021a)
	Diabetic rats	↓ Fat absorption by the body	Ben Bacha et al. (2018)
		↓ Fatty accumulation in the liver	Li et al. (2021b)
**Improving microbes in the gut**	Diabetic mouse	↓*Lactobacillus*, Dobrobacter, *Streptococcus*, Lactococcus, *Vibrio* thiobacillus	Hu et al. (2019)
		↑Bacteroidetes, *Haemophilus*, Ackermannia, Anaerobes	

## 8 Results

As one of the drugs commonly used in Chinese medicine for the treatment of diabetes, the role of mulberry leaves in regulating blood glucose has been well established in animal models of diabetes as well as in relevant clinical trials. From known studies, it has been found that the flavonoids, polyphenols, alkaloids and polysaccharides in mulberry leaves have good effects on the reduction of blood glucose in animal models of diabetes, and their therapeutic mechanisms include affecting the absorption and utilization of glucose, improving postprandial blood glucose and regulating insulin sensitivity in these direct ways, as well as indirectly reducing blood glucose by improving oxidative stress, improving lipid metabolism and balancing the intestinal environment. The results of the animal studies and human clinical trials presented in this article indicate that mulberry leaf can be used from multiple angles and targets to lower blood glucose and treat diabetes as a metabolic disease. We also investigated the safety of Mulberry Leaf in the treatment of diabetes and the safety evaluation showed that the side effects of Mulberry Leaf are very low compared to those of chemical drugs. These side effects disappear when the diabetic patient stops taking mulberry leaves. In the field of Chinese medicine, there are many important treatments for diabetes that are effective, as is the case with mulberry leaf, where the individual components or multiple components work in synergy to lower the sugar level, and this treatment coincides with the finishing theory of Chinese medicine. The treatment of diabetes takes a long time and during this time it is believed that mulberry leaves will exude its unique charm.

## 9 Discussion

Diabetes is a common metabolic disorder with a variety of causes and thus its pathogenesis is not yet fully understood. Most of the research on the treatment of diabetes with mulberry leaves has, as in the above article, addressed the difficult issue of diabetes from the whole part of the leaf to its individual components and their corresponding mechanisms. In addition to the above, researchers have been searching for other ways in which mulberry leaves can be used to combat diabetes. Mulberry leaves contain many trace elements such as iron, zinc, manganese and calcium (Brima and Siddeeg, 2022). Iron can scavenge free radicals in the body and increase the oxygen supply to pancreatic cells to facilitate the synthesis and release of insulin (Arredondo et al., 2011). Zinc promotes the crystallization of insulin to enhance insulin stability and strengthen the hypoglycemic effect of insulin (Yoshikawa et al., 2011). Manganese is a component of manganese superoxide dismutase necessary to reduce oxidative stress in mitochondria and this enzyme is also an antioxidant, protecting mitochondria and reducing their oxidative damage to protect islet β-cells from oxidative stress (Li and Yang, 2018). Calcium deficiency can adversely affect the secretory function of the cells, and insulin secretion is a significant calcium-mediated process, and when calcium is deficient the calcium balance inside and outside the cells is altered, and this altered balance can affect the normal secretion of insulin (Pittas et al., 2007). In addition to the above-mentioned beneficial trace metal elements in mulberry leaves, there are other nutrients such as ascorbic acid (vitamin C) and β-carotene that play a role in blood glucose control (Srivastava et al., 2006). Firstly, vitamin C is a good antioxidant that improves oxidative stress in the body, secondly, vitamin C can also restore essential fatty acid (EFA) metabolism by promoting the formation of prostaglandin E1 (PGE1), prostacyclin (PGI2) and endothelial nitric oxide (ENO), which are protective of pancreatic β-cells, and promote the formation of the antioxidant lipoxygen A4 (LXA4), and finally Vitamin C can also reduce inflammatory damage to pancreatic β-cells through its anti-inflammatory effects against tumour necrosis factor (TNF-α) and interleukin-6 (IL-6) produced by the body, which can be explained by the above pathways to combat diabetes and achieve good results (Das, 2019). Studies on β-carotene have shown that it is effective in alleviating the symptoms of many metabolic diseases such as type II diabetes, cardiovascular disease and obesity (Rodriguez-Concepcion et al., 2018; Mounien et al., 2019). In the fight against diabetes, β-carotene can alleviate insulin resistance and protect insulin receptors by effectively controlling oxidative stress, and indirectly regulate blood glucose by regulating lipid metabolism in the body (Marcelino et al., 2020).

In addition to its good anti-diabetic properties, mulberry leaf can also increase the efficacy of chemical drugs used in the treatment of diabetes by synergising them. By co-administration of mulberry leaf extract with metformin in diabetic rats, the researchers found that aqueous extracts of mulberry leaves administered at a quality of 25–100 mg/kg increased the hypoglycaemic effect of metformin in a dose-dependent manner. The pharmacokinetics showed a significant increase in the residence time of metformin in the body compared to the treatment group given metformin alone, which may be due to the inhibition of the transport protein OTC2 of metformin by the aqueous extract of mulberry leaves. The aqueous extract of mulberry leaves can inhibit the expression of OTC2 protein, which reduces the uptake of metformin by the kidneys and decreases the clearance of metformin through this pathway. metabolism (Shu et al., 2007; Geng et al., 2015; Huh et al., 2020).

In the treatment of diabetic patients we have to take into account not only the quality of the herbs but also their acceptability to the patient, including the smell and taste of the herbs. Researchers have mixed honey with mulberry leaves to make a mulberry leaf preparation which has greatly improved patient acceptance. There has been controversy regarding the safety of honey as an excipient for diabetic patients (Meo et al., 2017). Some argue that honey as a high carbohydrate can have an impact on the control of diabetic patients, while most argue that honey has more beneficial substances that can increase the body’s immunity to diabetes and its complications (Sahlan et al., 2020). When preparations were made with honey and mulberry leaves, it was found that the solubility of 11 components in mulberry leaves increased, including flavonoids and alkaloids, and *in vitro* and *in vivo* experiments showed that honey-mulberry leaf preparations were better at controlling blood glucose compared to single mulberry leaf preparations, which may be due to the higher solubility of the components in honey-mulberry leaves, but also to the fact that the nutrients in honey enhance the multifunctionality of human tissues and can help the body to better (Bai et al., 2021).

Although mulberry leaves have many benefits for diabetes, it is important to control the quality of the environment in which they are grown, as the quality of the herbs cannot be guaranteed if the environment is contaminated, which may aggravate the condition of diabetics and cause more serious pathological changes in the body. The above findings suggest that the multiple ways in which mulberry leaves can improve diabetes and its complications provide new research advances in the treatment of diabetes, and that the side effects of mulberry leaves are minimal compared to those of chemical drugs, which can improve the quality of life of diabetic patients. The investigation of the therapeutic mechanism of mulberry leaf for diabetes also provides a reference for the role of other Chinese herbal medicines in the treatment of diabetes and allows the Chinese medicine culture represented by mulberry leaf to be accepted by more people worldwide.
